# Detection of avian influenza virus: a comparative study of the in silico and *in vitro* performances of current RT-qPCR assays

**DOI:** 10.1038/s41598-020-64003-6

**Published:** 2020-05-21

**Authors:** Andrea Laconi, Andrea Fortin, Giulia Bedendo, Akihiro Shibata, Yoshihiro Sakoda, Joseph Adongo Awuni, Emilie Go-Maro, Abdelsatar Arafa, Ali Safar Maken Ali, Calogero Terregino, Isabella Monne

**Affiliations:** 1 Istituto Zooprofilattico Sperimentale delle Venezie, Viale dell’Università, 10, Legnaro, Padova 35020 Italy; 2 Exotic Disease Inspection Division, Laboratory Department, Animal Quarantine Service, Ministry of Agriculture, Forestry and Fisheries, Tokoname, Aichi Japan; 3 Laboratory of Microbiology, Faculty of Veterinary Medicine, Hokkaido University, Sapporo, Hokkaido Japan; 4 Accra Veterinary Laboratory, Accra, Ghana; 5 Laboratoire Central Vétérinaire de Lomé, Lomé, Togo; 6 Reference Lab for Veterinary Quality Control on Poultry Production, Animal Health Research Institute, Dokki, Giza 12618 Egypt; 7 Iran Veterinary Organization (IVO), Tehran, Tehran province Iran

**Keywords:** High-throughput screening, Microbiology techniques, Assay systems

## Abstract

Avian influenza viruses (AIV) are negative sense RNA viruses posing a major threat to the poultry industry worldwide, with the potential to spread to mammals, including humans; hence, an accurate and rapid AIV diagnosis is essential. To date AIV detection relies on molecular methods, mainly RT-qPCR directed against AIV M gene segment. The evolution of AIV represents a relevant issue in diagnostic RT-qPCR due to possible mispriming and/or probe-binding failures resulting in false negative results. Consequently, RT-qPCR for AIV detection should be periodically re-assessed both in silico and *in vitro*. To this end, a specific workflow was developed to evaluate in silico the complementarity of primers and probes of four published RT-qPCR protocols to their target regions. The four assays and one commercially available kit for AIV detection were evaluated both for their analytical sensitivity using eight different viral dilution panels and for their diagnostic performances against clinical specimens of known infectious status. Differences were observed among the tests under evaluation, both in terms of analytical sensitivity and of diagnostic performances. This finding confirms the importance of continuously monitoring the primers and probes complementarity to their binding regions.

## Introduction

Influenza A viruses (IAV) are enveloped negative-strand RNA viruses belonging to the family of *Orthomyxoviridae*. IAVs are important veterinary and human health pathogens, infecting many different avian and mammalian species worldwide^1^. Viruses of the *Influenza virus* A genus cause avian influenza (AI), a disease of great importance for animal health, both for the high mortality rate caused by some viral strains in domestic and wild birds and for public health implications due to their zoonotic potential. Based on the antigenic differences between the two surface glycoproteins hemagglutinin (HA) and neuraminidase (NA), AI viruses can be subtyped in 16 HA subtypes (H1–H16) and 9 NA subtypes (N1–N9)^2^. Remarkably, all have been isolated from avian species in most possible combinations. Influenza A viruses infecting poultry can be grouped based on their pathogenicity: highly pathogenic avian influenza (HPAI) viruses, which can cause flock mortality as high as 100%, and low pathogenic avian influenza (LPAI) viruses, which usually cause a milder or unapparent disease^2^. To date, only viruses typed as H5 and H7 have proved to be highly pathogenic in naturally infected poultry^1^.

Several diagnostic methodologies are currently available for the detection of AI infection, with virus isolation (VI) in eggs or in cell cultures universally recognized as the reference diagnostic standard. However, the application of such methods is mainly limited by the fact that they are not flexible to a sudden increase in demand, are not cost-effective, requires high biosafety standards and often a long processing time. For these reasons, in the recent past there has been a significant increase in the development and application of testing procedures for the detection of AI viral RNA. With the advent of molecular biology, several RT-PCR and RT-qPCR protocols have been developed for AIV detection and typing, proving to be rapid, specific and sensitive^3–6^. The approach to AIV diagnosis using molecular methods adopted in most laboratories has been based on the initial generic detection of AIV in clinical specimens, primarily by targeting the matrix (M) gene segment, followed by specific RT-qPCR tests for H5 and H7 subtype viruses^1^. The rationale behind the targeting of the M gene segment relies on the presence of regions sufficiently conserved among influenza A viruses of various species including avian ones and, hence, suitable for primers and probe selection. Despite several RT-PCR and RT-qPCR assays for generic AIV detection have been routinely and successfully used worldwide, some considerations are due. AIV, with its single-stranded negative-sense RNA genome, arranged into eight genomic segments, shows an intrinsic genetic instability^2^. This is mainly due to the error-prone nature of the virus replication machinery and to re-assortment during infection of a single host cell with two or more distinct AIV types, resulting in considerable genetic heterogeneity and evolutionary diversity^2^. Therefore, any molecular biology method should be periodically re-evaluated, on the ground that the sequence complementarity within the primers and probes binding regions might have changed, affecting the performances of the methods^7^.

In the present study, we compared the analytical sensitivity and the diagnostic performances of four published protocols for AIV M gene segment detection^6,8–10^ and a commercially available diagnostic kit.

The four published protocols were chosen on the grounds that they are routinely used in international and national avian influenza reference laboratories, while the commercial kit was included being the first PCR-based commercial kit for the detection of avian influenza licensed by the U.S. Department of Agriculture (USDA); furthermore, the kit was also evaluated by the former European Union AI-ND Reference laboratory (Animal and Plant Health Agency-Weybridge-UK) showing promising results^11^.

Prior *in vitro* evaluation, we performed a comprehensive in silico analysis to assess the level of identity between primers and probes and their target regions on a dataset based on AIV M gene segment sequences deposited from 2014 onwards.

## Materials and Methods

### Sequences download and multiple sequence alignment

AIV M gene segment sequences deposited from January 2014 onwards were downloaded from the Global Initiative on Sharing All Influenza Data (GISAID) webserver (https://platform.gisaid.org/). A total of 4088 sequences were aligned against primers and probes of the four published assays^6,8–10^ using MAFFT version 7 (https://mafft.cbrc.jp/alignment/software/). A unique multiple sequence alignment (MSA) analysis was performed for the assays developed by Spackman *et al*. (2002), Heine *et al*. (2015) and Hoffmann *et al*. (2016), these last two representing the improved versions of the former via the introduction of degenerated bases in the primers’ sequences (Table 1). An independent MSA analysis was performed for Nagy’s protocol, since primers and probe target a different gene region. Of notice, Nagy’s protocol used probe number 104 (UPL104), from the 165 Universal ProbeLibrary (Merck KGaA, Darmstadt, Germany), containing locked nucleic acids (LNA) to increase probe binding; probe sequence is public available^7^, although the manufacturer did not disclose LNAs positions, hence it was not possible to evaluate the genetic variability in respect to each LNA. No MSA analysis was performed for the commercial kit, as the manufacturer did not disclose any primers and probes sequences.

**Table 1:** List of the assays tested in the present study.

Protocol	FW (5′→3′)	Probe (5′→3′)	RV (5′→3′)	Position (M gene)	Size (bp)
Protocol 1	AGATGAGTCTTCTAACCGAGGTCG	TCAGGCCCCCTCAAAGCCGA	TGCAAAAACATCTTCAAGTCTCTG	25–124	99
Protocol 2	AGATGAGTCTTCTAACCGAGGTCG	TCAGGCCCCCTCAAAGCCGA	TGCAAA**R**ACATCTTCAAGT**Y**TCTG	25–124	99
Protocol 3	AGATGAG**Y**CTTCTAACCGAGGTCG	TCAGGCCCCCTCAAAGCCGA	TGCAAA**N**ACATC**Y**TCAAGTCTCTG	25–124	99
Protocol 4	GGCCCCCTCAAAGCCGA	GTGCCCAG	CGTCTACG**Y**TGCAGTCC	77–259	182
Commercial kit	Sequences not available. Supplier declares primers and probes target M and NP genes.				

For each assay the sequences of primers and probe (5′→3′), the target regions and the amplicon size are reported. The degenerated bases introduced in the primers sequences are highlighted in bold.

### *In silico* evaluation of nucleotide variability and genetic diversity

**Figure 1:**
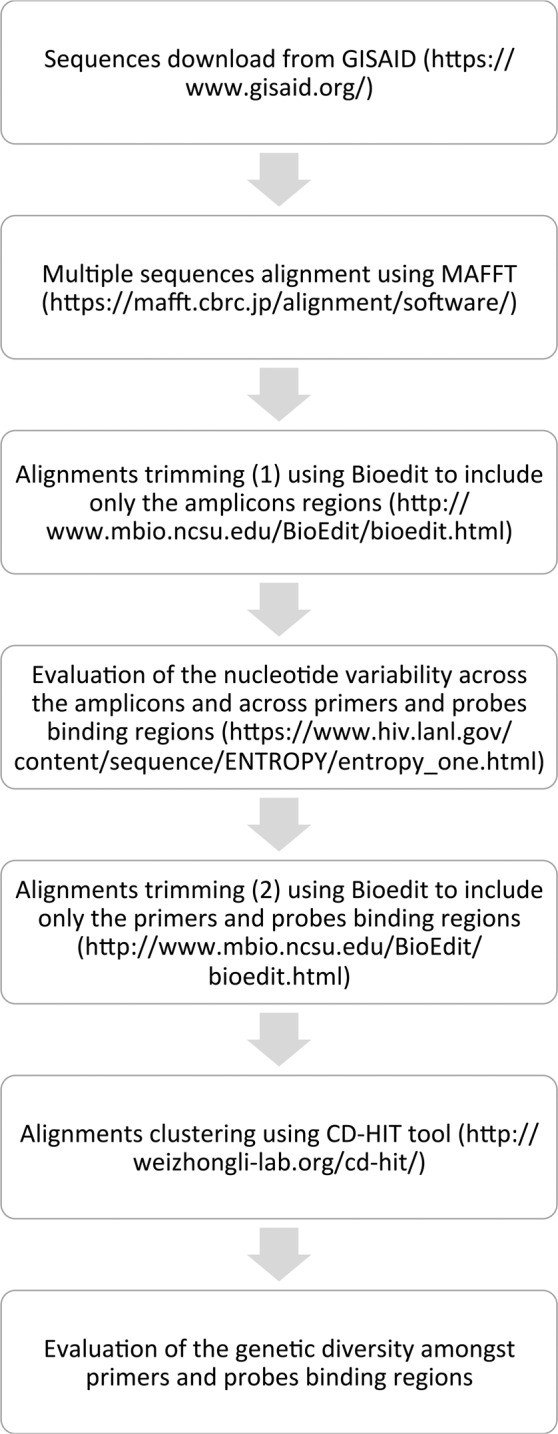
Schematic representation of the workflow used to evaluate the genetic diversity amongst primers and probes binding regions.

Using BioEdit (http://www.mbio.ncsu.edu/BioEdit/bioedit.html), the multiple sequence alignments were trimmed to include solely the sequence amplified by the assays, and the nucleotide variability within the amplicons was assessed using a web-based Shannon Entropy calculator (https://www.hiv.lanl.gov/content/sequence/ENTROPY/entropy_one.html). Sequences were further trimmed and concatenated, thus the resulting dataset contained merely the nucleotides complementary to primers and probes. The dataset were subsequently processed with cd-hit-est test of the CD-HIT Suite (http://weizhong-lab.ucsd.edu/cdhit_suite/cgi-bin/index.cgi?cmd=cd-hit-est)^12^ to cluster sequences that shared 100% identity, such as each cluster represents a unique primers-probe motif. A prototype sequence within each cluster was selected, and eventually each cluster was expanded to the original number of sequences, in order to evaluate the relevance of each cluster. Only clusters containing more than 30 sequences, or rather with an incidence >0.75%, were considered significant to assess the inclusivity of the assays, and hereafter referred as major clusters. Figure 1 depicts the workflow used for the evaluation of nucleotide variability and genetic diversity.

### Virus isolates and clinical samples

Eight viruses were selected according to the results of the in silico evaluation and used to assess the analytical sensitivity of the five assays (Table 2). Viruses were isolated and titrated by inoculation into the allantoic cavity of 9–11-day-old specific pathogens free (SPF) embryonated chicken eggs (ECEs). ECEs were candled daily up to 16 days of age and ECEs with dead embryos were transferred to 4 °C for 16–24 h prior harvesting of the allantoic fluid. Titre are expressed as the 50% embryonic infectious dose EID_50_/100 µl, as determined according to Reed and Muench^13^.

**Table 2:** List of the viral clusters identified and of the representative viruses used in the present study for the limit of detection (LoD) study.

Cluster	Size	Representative virus	Titre (EID50)	Primers and probe motifs (5′→3′)
A	870 seq	A/chicken/Japan/AQ-HE144/2015^20^ (H5N6)	10^8,5^	AGATGAGTCTTCTAACCGAGGTCG-TCAGGCCCCCTCAAAGCCGA-CAGA**A**ACTTGA**G**GATGT**G**TTTGCA
B	862 seq	A/chicken/Italy/1670/2015 (H7N2)	10^7,5^	AGATGAGTCTTCTAACCGAGGTCG-TCAGGCCCCCTCAAAGCCGA-CAGAGACTTGAAGATGT**C**TTTGCA
C	758 seq	Not available in our repository	N/A	AGATGAGTCTTCTAACCGAGGTCG-TCAGGCCCCCTCAAAGCCGA-CAGAGACTTGAAGATGT**G**TTTGCA
D	286 seq	A/turkey/Italy/17VIR576–11/2017 (H5N8)	10^8,3^	AGATGAGTCTTCTAACCGAGGTCG-TCAGGCCCCCTCAAAGCCGA-CAGAGACTTGAAGATGT**C**TTTG**T**A
E	271 seq	Not available in our repository	N/A	AGATGAGTCTTCTAACCGAGGTCG-TCAGGCCCCCTCAAAGCCGA-CAGAGACTTGAAGATGT**A**TTTGCA
F	177 seq	A/chicken/Togo/17RS1021-1/2017 (H5N1)	10^8,5^	AGATGAGTCTTCTAACCGAGGTCG-TCAGGCCCCCTCAAAGCCGA-CAGA**A**ACTTGA**G**GATGT**A**TTTGCA
G	124 seq	A/chicken/Ghana/18VIR1513-11/2017 (H9N2)	10^8,5^	AGATGAGTCTTCTAACCGAGGTCG-TCAGGCCCCCTCAAAGCCGA-CAGAGACTTGA**G**GATGTTTTTGCA
H	93 seq	A/chicken/Italy/1279/99_L2/2018 (H7N1)	10^7,5^	AGATGAGTCTTCTAACCGAGGTCG-TCAGGCCCCCTCAAAGCCGA-CAGAGACTTGAAGATGTTTTTGCA
I	66 seq	A/chicken/Egypt/1709-6/2008 (H5N1)	10^7,6^	AGATGAGTCTTCTAACCGAGGTCG-TCAGGCCCCCTCAAAGCCGA-CAGA**A**ACTTGAAGATGT**C**TTTGCA
L	45 seq	Not available in our repository	N/A	AGATGAGTCTTCTAACCGAGGT**T**G-TCAGGCCCCCTCAAAGCCGA-CAGAGACTTGAAGATGTTTTTGCA
M	39 seq	A/chicken/Iran/10VIR854-4/2009 (H9N2)	10^7,8^	AGATGAG**C**CTTCTAACCGAGGTCG-TCAGGCCCCCTCAAAGCCGA-CAGAGACTTGAAGATGTTTTTGCA

For each representative virus the titre (EID_50_/100 µl) and the unique primers and probe motif are reported. To highlight the genetic variability amongst the viral cluster, the nucleotide changes with respect to the primers and probe of protocol 1 are reported in bold.

A total of 152 clinical samples of known infectious status, were used for the comparison of the diagnostic performances of the five assays. More specifically, 79 were AIV positive field samples of European (n = 52), African (n = 22) and Asian (n = 5) origin sent to our laboratory for diagnostic purpose as national, European and OIE/FAO reference laboratory (pre-typed either by classical methods after virus isolation or by sequencing) and 73 AIV negative samples. These latter represented isolates of other avian pathogens, both viruses and bacteria, as well as true negative samples obtained from SPF chickens. True negative samples obtained from SPF chickens represent specimens collected during prior animal experiments conducted in our institution; animals’ manipulation was conducted in accordance with the Decree of the Ministry of Health n. 26 of 4 March 2014 on the protection of animals used for scientific purposes, implementing Directive 2010/63/EU. Specimens included tracheal/oropharyngeal and cloacal swabs, lungs, tracheas, intestines, brains, faeces, tissues homogenates, allantoic fluids and FTA cards.

### Nucleic acids isolation

Total nucleic acids were extracted using QIAsymphony DSP Virus/Pathogen Midi kit (Qiagen, Hilden, Germany), in combination with the automated system QIAsymphony SP (Qiagen, Hilden, Germany). Isolation of the nucleic acids was performed following the manufacturer’s recommendations and to each sample an internal process control (Intype IC-RNA, Qiagen, Hilden, Germany) was added.

### RT-qPCR assays

#### Protocol 1 - Spackman *et al*., 2002

The assay, hereafter referred to as protocol 1 for sake of clarity, was carried out using OneStep RT-PCR kit (Qiagen, Hilden, Germany) in a final mastermix of 25 µl containing 0.3 µM of each primer, 0.1 µM of the probe, 2 µl of IC mix and 5 µl of nucleic acid. The following thermoprofile was used: initial step at 50 °C for 30 min, 95 °C for 15 min, 40 cycles at 94 °C for 45 sec and 60 °C for 45 sec.

#### Protocol 2 - Hoffmann *et al*., 2016

The assay, hereafter referred to as protocol 2, was carried out using AgPath-ID™ One-Step RT-PCR kit (Applied Biosystems, Foster City, CA) in a final mastermix of 25 µl containing 1.6 µM of the forward primer, 1.2 µM of each reverse primer, 0.2 µM of probe, 2 µl of IC mix and 5 µl of nucleic acid. The following thermoprofile was used: initial step at 45 °C for 10 min, 95 °C for 10 min, 40 cycles at 95 °C for 15 sec, 56 °C for 20 sec, 72 °C 30 sec.

#### Protocol 3 - Heine *et al*., 2015

The assay, hereafter referred to as protocol 3, was carried out using AgPath-ID™ One-Step RT-PCR kit (Applied Biosystems, Foster City, CA) in a final mastermix of 25 µl containing 0.9 µM of the forward primer, 0.225 µM of each reverse primer, 0.25 µM of probe, 2 µl of IC mix and 5 µl of nucleic acid. The following thermoprofile was used: initial step at 45 °C for 10 min, 95 °C for 10 min, 45 cycles at 95 °C for 15 sec and 60 °C for 45 sec.

#### Protocol 4 - Nagy *et al*., 2010

The assay, hereafter referred as protocol 4, was carried out using QuantiFast Probe RT-PCR kit (Qiagen, Hilden, Germany) in a final mastermix of 25 µl containing 0.6 µM of each primer, 0.212 µM of the Universal Probe 104 (Merck KGaA, Darmstadt, Germany), 2 µl of IC mix and 5 µl of nucleic acid. The following thermoprofile was used: initial step at 50 °C for 20 min, 95 °C for 5 min, 45 cycles at 95 °C for 10 sec, 60 °C for 30 sec, 72 °C 10 sec. Furthermore, as stated by Nagy *et al*., the 60 °C–72 °C ramp temperature was decreased to 1 °C/s.

#### Commercial kit - VetMAX-Gold AIV Detection Kit

The assay, hereafter referred as commercial kit, was carried out following the manufacturer’s recommendation (Thermo Fisher Scientific) in a final mastermix of 25 µl containing 8 µl of nucleic acid and 2 µl of IC mix. The following thermoprofile was used: initial step at 48 °C for 10 min, 95 °C for 10 min, 40 cycles at 95 °C for 15 sec, 60 °C for 45 sec.

RT-qPCRs were carried out on CFX 96 Real-Time PCR Detection Systems (Biorad, Munich, Germany). Reaction mix of protocols 1 and 4 was prepared following the recommendations of the former Avian Influenza Community Reference Laboratory (Animal and Plant Health Agency, UK) (https://science.vla.gov.uk/flu-lab-net/docs/pub-protocol-ai-vi493.pdf), while for the remaining assays the authors’ or the manufacturer’s recommendations were followed. The only deviation from the recommended protocols was the addition of 2 µl IC mix to each reaction.

### Assays performances and limit of detection study

The analytical sensitivity of each RT-qPCR assay was assessed using 10-fold serial dilutions of eight titrated AI viruses selected as representative of the M gene segment clusters identified through the in silico evaluation (Table 2). Each dilution was prepared in triplicate and tested by each assay on the same day. The limit of detection (LoD) was defined as the highest dilution at which all replicates tested positive (cycle threshold < 36).

### Diagnostic sensitivity and specificity study

A total of 152 clinical samples of known infectious status were tested in duplicate by each of the five assays to assess their diagnostic performances. A sample was considered positive when both the replicates produced a sigmoidal amplification curve (Ct < 36). Sample size, together with diagnostic sensitivity (DSe) and specificity (DSp) of all the assays under evaluation, were established as recommended in the OIE Terrestrial Manual 2018 – Principal and methods of validation of diagnostic assay for infectious diseases^14^.

### Statistical analysis

Two-way analyses of variance (ANOVA) with Tukey’s HSD post hoc test was performed to assess whether the analytical sensitivity was statistically different between the assays.

MacNemar’s Chi-square test was used to confirm the null hypothesis that diagnostic sensitivity and specificity were equal among assays, while Kappa values (ƙ) were also calculated as a measure of overall agreement between assays, with values categorized as slight (ƙ < 0.2), fair (0.2 ≤ ƙ≤0.4), moderate (0.4 ≤ ƙ ≤ 0.6), substantial (0.6 ≤ ƙ ≤ 0.8) or almost perfect (ƙ >  0.8) agreement^15^.

Two-way ANOVA, MacNemar’s Chi square and Kappa values were calculated using GraphPad software for categorical data (http://graphpad.com/quickcalcs/).

## Results

### Evaluation of nucleotide and genetic variability between and within primers and probes binding regions

**Figure 2:**
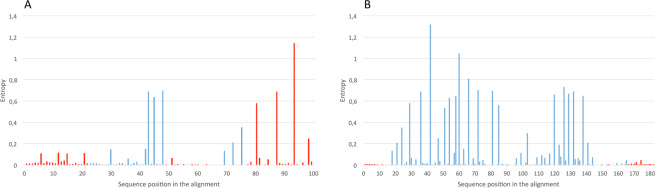
Entropy plots. (**A**) The nucleotide variation amongst the 99 bp fragment of the AIV M gene segment (positions 25–124) amplified by protocols 1, 2 and 3 shown as entropy. (**B**) The nucleotide variation amongst the 182 bp fragment of the AIV M gene segment (position 77–259) amplified by protocol 4 shown as entropy. The height of each column is proportional to the nucleotide variation at the given position. In red the primers and probes binding regions.

First, the sequences were trimmed to span the amplicons of each assay; entropy plots summarize the nucleotide variability at each position of the amplicons. The entropy plot of the amplicons of protocols 1, 2 and 3 (Fig. 2A) revealed high variability at three nucleotide positions within the sequence targeted by their reverse primers; the analysis showed two transitions at position 80 and 87 of the amplicons, G to A and A to G respectively, while at position 93 a combination of all the four bases with similar frequency was observed. The degenerated bases in the reverse primers of protocols 2 and 3 match the nucleotide diversity at positions 80 and 93, and 87 and 93, respectively. The binding regions of primers and probe of protocol 4 showed almost no variability (Fig. 2B).

The alignments were further trimmed in order to merely consider the regions complementary to primers and probes; the obtained dataset were clustered using CD-HIT tool, with the aim of identifying unique primers-probe motifs within the AIV M gene segment genetic diversity. The datasets for protocols 1, 2 and 3 were characterized by eleven major clusters (>30 sequences) accounting for 3591 AIV M gene segment sequences, or rather 87,8% of AIV genetic diversity within the primers and probes binding regions. The dataset obtained for protocol 4 contains only one major cluster, showing 100% identity within the primers and probes binding regions and accounting for 3918 AIV M gene segment sequences (95.8%).

Based on these datasets, it was possible to identify the eight AIV strains used in the limit of detection study, which were representative of the genetic diversity of 62% of the AIVs circulating worldwide since 2014 within the target region of the primers and probes of the four published protocols.

### Limit of detection of the RT-qPCR assays

A dilution panel for each virus was tested in triplicate by the five assays. The LoD was defined as the highest dilution at which all replicates tested positive (Ct < 36). The LoD of the assays varies among the dilution panels; however, protocols 2 and 3 and the commercial kit proved to be consistently the most sensitive assays (Table 3). Protocol 3 and the commercial kit showed the best analytical sensitivity in seven out of eight dilution panels, with the exception of dilution panels D and G, while protocol 2 was second best only when tested against dilution panels G and M. Protocol 4 matched the best analytical sensitivity in four out of eight dilutions panels. Sensitivity of protocol 1 was significantly the lowest among all the dilution panels, with the exception of dilution panel B (Table 3).

**Table 3:** Limit of detection (LoD) study. LoDs are reported as viral titre in EID_50_/100 µl.

Cluster	Protocol 1			Protocol 2			Protocol 3			Protocol 4			Commercial kit		
	LoD	Mean Ct	SD	LoD	Mean Ct	SD	LoD	Mean Ct	SD	LoD	Mean Ct	SD	LoD	Mean Ct	SD
A	10^2,5^	34,76	0,35	10^1,5^	33,53	0,15	10^1,5^	33,46	0,24	10^1,5^	34,95	0,59	10^1,5^	33,39	0,18
B	10^1,5^	35,38	0,29	10^1,5^	31,87	0,03	10^1,5^	31,75	0,22	10^1,5^	34,59	0,38	10^1,5^	32,57	0,08
D	10^3,3^	35,03	0,24	10^2,3^	35,90	0,31	10^3,3^	32,76	0,19	10^4,3^	32,38	0,26	10^2,3^	35,38	0,27
F	10^3,5^	32,63	0,48	10^1,5^	33,91	0,52	10^1,5^	35,63	0,05	10^1,5^	35,23	0,19	10^1,5^	33,55	0,12
G	10^3,5^	32,45	0,20	10^2,5^	35,42	1,28	10^1,5^	35,19	0,37	10^2,5^	31,95	0,13	10^2,5^	35,62	0,10
H	10^2,5^	32,76	0,22	10^1,5^	34,14	0,13	10^1,5^	35,84	0,41	10^2,5^	33,28	0,57	10^1,5^	33,90	0,16
I	10^3,6^	35,06	0,32	10^1,6^	33,56	0,11	10^1,6^	34,67	0,34	10^2,6^	32,68	0,56	10^1,6^	34,12	0,06
M	10^1,8^	33,44	0,08	10^1,8^	30,89	0,15	10^0,8^	35,98	0,81	10^0,8^	35,77	0,20	10^0,8^	33,81	0,41

Ct values shown represent the mean of three independent replicates.

Statistical analysis of Ct values at the same viral titre at optimal baseline and threshold settings for all the assays showed a significant difference (p < 0.05) between Ct values of protocols 1 and those of the remaining assays amongst all the dilution panels, with the exception of dilution panel D (Table 4).

**Table 4:** Statistical analyses.

	Diagnostic performances			Analytical sensitivity							
	K	χ DSe	χ DSp	Cluster A	Cluster B	Cluster D	Cluster F	Cluster G	Cluster H	Cluster I	Cluster M
Protocol 1 vs Protocol 2	0,90	1,73E-05	1,000	0,001	0,001	0,004	0,001	2,43E-04	0,004	1,05E-07	3,67E-04
Protocol 1 vs Protocol 3	0,91	1,86E-12	1,000	0,001	0,002	0,027	0,002	8,90E-09	0,004	3,74E-06	1,27E-05
Protocol 1 vs Protocol 4	0,87	0,396	0,172	3,71E-06	0,109	0,022	0,002	2,22E-08	0,330	5,42E-05	1,72E-06
Protocol 1 vs Commercial kit	0,90	6,39E-138	1,000	8,64E-08	2,52E-06	0,612	6,77E-06	1,98E-04	9,63E-05	2,16E-07	3,01E-08
Protocol 2 vs Protocol 3	0,96	0,314	1,000	0,016	0,208	0,099	0,001	2,55E-05	0,002	2,41E-04	0,071
Protocol 2 vs Protocol 4	0,92	0,089	0,172	1,11E-07	0,001	0,005	0,001	1,33E-04	1,14E-04	3,64E-04	0,069
Protocol 2 vs Commercial kit	0,97	1,83E-09	1,000	0,005	0,003	0,338	0,323	0,002	0,028	1,15E-04	0,114
Protocol 3 vs Protocol 4	0,89	0,034	0,172	1,75E-04	0,001	0,008	0,004	3,94E-07	1,12E-04	0,002	0,084
Protocol 3 vs Commercial kit	0,99	0,004	1,000	0,028	0,013	0,431	3,72E-07	2,75E-08	0,125	0,214	0,001
Protocol 4 vs Commercial kit	0,90	8,74E-78	0,172	0,003	0,002	0,704	3,57E-04	8,30E-08	7,85E-04	0,005	2,19E-04

Kappa (ƙ) values were calculated to assess the assays level of agreement. Values were categorized as follows: slight (ƙ < 0.2), fair (0.2 ≤ ƙ ≤ 0.4), moderate (0.4 ≤ ƙ ≤ 0.6), substantial (0.6 ≤ ƙ ≤ 0.8) or almost perfect (ƙ > 0.8) agreement. Chi-square test was used to discount the null hypothesis that diagnostic sensitivity (DSe) and diagnostic specificity (DSp) were equal amongst the assays. To assess whether the analytical sensitivity was statistically different among the assays in respect to each cluster, we performed a two-way analysis of variance (ANOVA) followed by Tukey’s honestly significant difference (HSD) post hoc test. Results are expressed as p-value; a p-value<0.05 was considered significant.

### Diagnostic sensitivity and specificity of the RT-qPCR assays

To assess and compare the diagnostic performances of the five assays, 152 samples of known infectious status were tested in parallel on the same RNA extracts. Details of the diagnostic performances of the five assays are reported in Table 5. The best performances for AIV M gene segment detection from clinical specimens were observed for the commercial kit (DSe = 100%), followed by protocol 3 (DSe = 98,78%), protocol 2 (DSe = 97.47%) and protocol 4 (DSe = 92.41%), with protocol 1 performing the least (DSe = 89.87%). The diagnostic sensitivity of the commercial kit differs (p < 0.05) from those of the remaining assays (Table 4), with the exception of protocol 3, which confirms that these two assays yielded the best performances for AIV detection from clinical specimens. Similarly, the diagnostic sensitivity of protocol 1 was statistically different (p < 0.05) from that of the other assays (Table 4), with the exception of protocol 4, a data confirming the lower diagnostic sensitivity of the assay.

**Table 5:** Detection results of RT-qPCR assays using 152 samples of known infectious status.

Viruses			Protocol 1		Protocol 2		Protocol 3		Protocol 4		Commercial kit	
Origin	HA subtype	Clade	Results	%	Results	%	Results	%	Results	%	Results	%
Africa	H9	N/A	7/7	100,00	7/7	100,00	7/7	100,00	6/7	85.71	7/7	100,00
	H5	2.3.2.1c	8/9	88,89	9/9	100,00	8/9	88,89	9/9	100,00	9/9	100,00
		2.3.4.4b	5/6	83,33	6/6	100,00	6/6	100,00	6/6	100,00	6/6	100,00
Eurasia	H11	N/A	2/2	100,00	2/2	100,00	2/2	100,00	1/2	50,00	2/2	100,00
	H5	2.3.4.4b	43/48	89,58	46/48	95,83	48/48	100,00	44/48	91,67	48/48	100,00
		2.3.4.4c	2/2	100,00	2/2	100,00	2/2	100,00	2/2	100	2/2	100,00
	H4	N/A	0/1	0,00	1/1	100,00	1/1	100,00	1/1	100,00	1/1	100,00
	H6	N/A	1/1	100,00	1/1	100,00	1/1	100,00	1/1	100,00	1/1	100,00
	H7	N/A	3/3	100,00	3/3	100,00	3/3	100,00	3/3	100,00	3/3	100,00
Diagnostic sensitivity			71/79	89,87	77/79	97,47	78/79	98,73	73/79	92,41	79/79	100,00
Diagnostic specificity (non-AIV)			73/73	100,00	73/73	100,00	73/73	100,00	71/73	97,26	73/73	100,00

Samples were grouped accordingly to origin, HA subtype and clade (if applicable). Diagnostic sensitivity and diagnostic specificity are reported.

Diagnostic specificity (DSp) ranged from a value of 97,2% for protocol 4 to 100% for the remaining assays; no statistical difference (p > 0.05) in terms of diagnostic specificity was observed between the assays (Table 4).

Kappa (ƙ) values were calculated as a measure of overall agreement among the assays, which proved to be almost perfect (ƙ > 0.80) (Table 4).

## Discussion

Avian influenza viruses exhibit a significant degree of genetic variability; this might lead to diagnostic failures of molecular tests when applied to mutated or new emerging viruses, meaning that a constant monitoring of the efficacy of molecular protocols available is uttermost necessary even when directed towards parts of the viral genome conventionally considered more stable. To our knowledge, this is the first large-scale in silico and *in vitro* evaluation of the RT-qPCR assays for the detection of AIV from different avian specimens. With the purpose of obtaining useful data on the performances of the assays in use in national and international reference laboratories, we compared the analytical sensitivity and the diagnostic performances of four published protocols^6,8–10^ and one licensed commercial kit (VetMAX-Gold AIV Detection Kit, Thermo Fisher Scientific).

When tested against a panel of 152 clinical samples of known infectious status, the five assays yielded comparable results (ƙ > 0.80); however, despite the overall satisfactory level of correlation, discrepancies were observed that deserve further discussion. Protocols 1 and 4 showed lower diagnostic sensitivity (DSe = 89.87% and DSe = 92.41 respectively), giving false negative results when analysing samples which produced a positive signal at late amplification cycles (Ct > 30) when tested with the other protocols. The results observed for protocol 1 are consistent with the low analytical sensitivity detected throughout all the tested dilution panels. It is important to notice that this protocol has been developed in early 2000; hence, primers and probe were designed in accordance with the M gene segment sequences of the viruses circulating at the time. Not surprisingly, the in silico analyses performed on AIVs circulating from 2014 onwards showed a certain lack of identity within their binding regions. The lower complementarity is likely responsible for the limited performances of this assay in comparison to the others, which benefit from an up to date design of primers and probes; to support, in the reverse primers of protocols 2 and 3 degenerated bases matching the high variability observed within their target region were introduced. As a whole, the lower analytical sensitivity and the poor performances observed in the diagnostic setting suggest that protocol 1, due to the continuous evolution of the virus, might not represent anymore the ideal assay for AIV detection. Remarkably, a lack of identity of the reverse primer of protocol 1 was previously observed in respect to the pandemic (H1N1) 2009 influenza virus and swine influenza A viruses (SIVs), leading to the development of an improved version of protocol 1 via the employment of a reverse primer specifically designed to match the genetic diversity of the pandemic and swine influenza viruses^16^. The employment of this new reverse primer might improve the diagnostic performance of protocol 1 towards AIV detection; however, in silico analysis using the dataset and the workflow presented in this study shows that these two reverse primers, even when used in combination, match only partially the genetic diversity of recent AIVs (supplementary table 1). On the contrary, in silico analysis confirmed the superior complementarity between primers and probe of protocol 4 and their target regions, suggesting that the reasons explaining the lower DSe must be ascribable to other factors. Protocol 4 amplifies a gene portion almost double in size in comparison to the other assays, possibly negatively affecting its efficiency and causing the low DSe observed. This hypothesis seems to be corroborated by the data gained during the limit of detection study, as protocol 4 showed an overall lower sensitivity in comparison to the best performing assays and in accordance with previous observation^11^. Another factor influencing the sensitivity of RT-qPCR assay is RNA integrity; considering the large gene portion amplified by protocol 4, we speculate that the diagnostic performances of this assays might be more affected by low quality RNA than the other protocols.

The assays of recent development, protocols 2, 3 and the commercial kit, showed the best analytical and diagnostic performances, underlying the importance of monitoring AIV M gene segment evolution and the need to update primers and probes sequences in relation to their binding regions. This need has already been proved for protocols aiming to type AIV^5,8,17^, or rather protocols targeting the HA gene segment known to rapidly evolve. By comparing the performance of five different RT-qPCR assays, our study clearly demonstrates that the same applies to the molecular assays for AIV detection, or rather to protocols targeting the conserved M gene segment^18,19^. To this aim, the in silico evaluation workflow proposed here represents a useful and user-friendly tool for the assessment of primers and probes complementary to their binding regions. Unfortunately, the unavailability of primers and probes sequences of the commercial kit implies having to rely on the manufacturer for such monitoring activity. To some extent, the same applies to protocol 4, as the manufacturer does not disclose the positions of the locked nucleic acids in the UPL104 probe, limiting the monitoring activity. One further concern about the UPL104 probe used in protocol 4 is related to its stability, as some level of degradation leading to false positive results has been observed while performing this study (data not shown). Implementation of good laboratory practice (e.g. aliquot UPL104) should be sufficient to avoid UPL104 degradation; however, the authors recommend extra carefulness in the storage and use of the probe.

The development of assays based on multiple targets is likely to reduce the risk of yielding false negative results; the in silico workflow described in this study, if applied to the whole M gene segment and/or other AIV genes, may lead to the identification of other conserved regions suitable for the implementation of such assays for generic AIV detection.

To conclude, our study confirms the importance of continuously monitoring the performances of the assays for AIV detection, both in silico and *in vitro*, as the emergence of new strains containing mutations within primers and probes binding regions might strongly affect the positive outcome of the test.

## Supplementary information

Supplementary Dataset 1.

## Data Availability

The datasets generated and analysed during the current study are available from the corresponding authors on reasonable request.

## References

[1] Avian Influenza (Infection with avian influenza viruses). OIE Terr. Man. 2015 - Chapter 2.3.4 (2015).

[2] Saurez, D. E. Common aspects of animal influenza. *Anim. Influ*. 3–29 (2017).

[3] Monne I (2008). Development and validation of a one-step real-time PCR assay for simultaneous detection of subtype H5, H7, and H9 avian influenza viruses. J. Clin. Microbiol..

[4] Slomka MJ (2007). Identification of Sensitive and Specific Avian Influenza Polymerase Chain Reaction Methods Through Blind Ring Trials Organized in the European Union. Avian Dis..

[5] Spackman E, Ip HS, Suarez DL, Slemons R, Stallknecht D (2008). Analytical validation of a real-time RT-PCR test for Pan-American lineage H7 subtype avian influenza viruses. J. Vet. Diagnostic Investig..

[6] Spackman E (2002). Development of a real-time reverse transcriptase PCR assay for type A influenza virus and the avian H5 and H7 hemagglutinin subtypes. J. Clin. Microbiol..

[7] Nagy A, Jiřinec T, Jiřincová H, Černíková L, Havlíčková M (2019). In silico re-assessment of a diagnostic RT-qPCR assay for universal detection of Influenza A viruses. Sci. Rep..

[8] Heine HG (2015). Detection of highly pathogenic zoonotic influenza virus H5N6 by reverse-transcriptase quantitative polymerase chain reaction. Virol. J..

[9] Hoffmann B, Hoffmann D, Henritzi D, Beer M, Harder TC (2016). Riems influenza a typing array (RITA): An RT-qPCR-based low density array for subtyping avian and mammalian influenza a viruses. Sci. Rep..

[10] Nagy A (2010). Development and evaluation of a one-step real-time RT-PCR assay for universal detection of influenza A viruses from avian and mammal species. Arch. Virol..

[11] Reid, S. *et al*. Evaluation of the VetmaxTM-Gold AIV detection kit and VetmaxTM-Gold SIV detection kit for detection of avian and swine influenza viruses. in 5TH CONGRESS OF THE EUROPEAN ASSOCIATION OF VETERINARY LABORATORY DIAGNOSTICIANS 14 – 17 OCTOBER, 2018, MCE BUSINESS AND CONFERENCE CENTRE, BRUSSELS, BELGIUM 105 (2018).

[12] Li W, Godzik A (2006). Cd-hit: A fast program for clustering and comparing large sets of protein or nucleotide sequences. Bioinformatics.

[13] Reed LJ, Muench H (1938). A simple method of estimating fifty per cent endpoints. Am. J. Hygene.

[14] Principles and methods of validation of diagnostic assays for infectious diseases. OIE Terr. Man. 2018 - Chapter 1.1.6. (2018).

[15] Dohoo, I. R., Martin, S. W. & Stryhn, H. *Veterinary Epidemiologic Research*. (VER, Incorporated, 2009).

[16] Slomka MJ (2010). Real time reverse transcription (RRT)-polymerase chain reaction (PCR) methods for detection of pandemic (H1N1) 2009 influenza virus and European swine influenza A virus infections in pigs. *Influenza Other Respi*. Viruses.

[17] Slomka MJ (2007). Validated H5 Eurasian Real-Time Reverse Transcriptase–Polymerase Chain Reaction and Its Application in H5N1 Outbreaks in 2005–2006. Avian Dis..

[18] Ito T, Gorman OT, Kawaoka Y, Bean WJ, Webster RG (1991). Evolutionary analysis of the influenza A virus M gene with comparison of the M1 and M2 proteins. J. Virol..

[19] Widjaja L, Krauss SL, Webby RJ, Xie T, Webster RG (2004). Matrix Gene of Influenza A Viruses Isolated from Wild Aquatic Birds: Ecology and Emergence of Influenza A Viruses. J. Virol..

[20] Shibata A (2018). Isolation and characterization of avian influenza viruses from raw poultry products illegally imported to Japan by international flight passengers. Transbound. Emerg. Dis..

